# Fifteen Years of Family Medicine Residency Training in the United Arab Emirates: A Mixed-Methods Evaluation of Program Evolution, Educational Outcomes, and Lessons Learned

**DOI:** 10.7759/cureus.111453

**Published:** 2026-06-24

**Authors:** Hayfa H AlAli, Zainab M Alameeri

**Affiliations:** 1 Primary Healthcare, Emirates Health Services, Dubai, ARE

**Keywords:** educational outcomes, family medicine residency, mixed methods research, post-graduate medical education, workforce development

## Abstract

Background

Family medicine plays a central role in strengthening primary healthcare systems and addressing evolving population health needs, particularly in regions undergoing rapid healthcare transformation. Over the past 15 years, the Emirates Health Services (EHS) Family Medicine Residency Program in the United Arab Emirates (UAE) has aimed to develop a competent family medicine workforce capable of delivering comprehensive, community-oriented, and patient-centered care.

Methods

This sequential mixed-methods study evaluated the evolution and perceived educational outcomes of the EHS Family Medicine Residency Program between 2009 and 2025. Quantitative data were collected using a structured survey administered to program alumni and current fourth-year residents (R4), while qualitative data were obtained through focus group discussions with residents, alumni, and faculty members. Quantitative analyses included descriptive statistics, non-parametric subgroup comparisons, and Spearman correlation analyses. Qualitative data were analyzed using inductive thematic analysis. Findings were integrated during interpretation.

Results

Of 71 eligible participants, 64 completed the survey (response rate: 90.1%), including 56 alumni and eight current R4 residents. Overall satisfaction with the residency program was high, with a median satisfaction score of 4.55/5.00. Faculty teaching, supervision, and academic day activities received the highest ratings, whereas hospital-based training and work-life balance demonstrated comparatively greater variability. Median perceived competency and preparedness scores were 4.36/5.00 and 4.37/5.00, respectively. Satisfaction demonstrated moderate positive correlations with perceived competency (Spearman ρ = 0.60, p < 0.0001) and preparedness for independent practice (ρ = 0.64, p < 0.0001). Outcome scores differed across post-residency duration groups, with alumni more than 10 years post-residency reporting the highest satisfaction, perceived competency, and preparedness scores. Qualitative findings suggested substantial program evolution toward a more structured, learner-centered, and competency-oriented training model, with strengths in academic-clinical integration, supervision, assessment systems, ambulatory learning, and resident professional development. Persistent challenges included workload pressures, administrative burden, and variability in hospital-based supervision.

Conclusion

Over the past 15 years, the EHS Family Medicine Residency Program has undergone substantial development toward a more structured and competency-oriented training model. Participants generally perceived the program positively in relation to supervision, educational experiences, preparedness for independent practice, and professional development. Ongoing operational and workload-related challenges warrant continued program refinement and longitudinal evaluation.

## Introduction

Family medicine (FM) is a medical specialty that offers continuous, comprehensive, and regular healthcare support to individuals and families across all ages, genders, and diseases [1]. Family medicine emphasizes a patient-centered approach that focuses on building long-term relationships with patients and addressing health issues within the context of their families and communities. Core principles of family medicine include continuity of care, comprehensive scope, patient-centeredness, and community orientation [2].

FM is particularly crucial in regions where healthcare systems are evolving and where there is an increasing demand for holistic, preventative, and community-based care. In such contexts, family medicine residency programs not only support standardization and professionalization of primary care delivery but also help address the growing burden of chronic diseases, aging populations, and healthcare access inequities [3]. This is especially relevant in the United Arab Emirates (UAE), where rapid socioeconomic development has transformed the healthcare landscape over the past two decades. In the northern emirates of the UAE, where the family physician workforce has historically been limited, the establishment and development of a family medicine residency training program represents an important development in local healthcare reform and workforce capacity building.

Strong primary care systems anchored in FM are consistently associated with improved population health outcomes, enhanced preventive care delivery, reduced avoidable hospitalizations, greater healthcare equity, and more cost-effective healthcare utilization. In addition to providing comprehensive and continuity-based care, FM plays an important role in integrating individual patient management with broader public health priorities and community health needs. Consequently, strengthening family medicine training has become an increasingly important global health workforce strategy, particularly in healthcare systems seeking to improve primary care capacity and progress toward universal health coverage [4, 5].

International organizations, including the World Health Organization (WHO) and the World Organization of Family Doctors (WONCA), have emphasized the importance of strengthening FM and primary healthcare systems through investment in structured postgraduate training, workforce development, and community-oriented models of care [6]. Accordingly, evaluating how residency programs evolve over time may provide important insights into educational effectiveness, workforce development, and healthcare system strengthening [6]. The published literature from the UAE has focused on program establishment, curricular descriptions, or short-term learner outcomes rather than longitudinal program evolution and perceived educational impact [7].

In the UAE, and specifically the northern emirates, the implementation of FM residency training has been both timely and strategically important. The northern emirates have historically faced disparities in healthcare infrastructure and workforce distribution when compared to metropolitan hubs like Dubai and Abu Dhabi [8]. In response, local health authorities and academic institutions initiated a family medicine residency program aimed at producing a new generation of physicians equipped to serve the diverse needs of these communities [7].

The Emirates Health Services (EHS) family medicine residency program was established in 2009 and accredited by the Arab Board for Medical Specialization as a four-year training program. The program aimed to prepare family physicians capable of delivering comprehensive, patient-centered care through continuous quality improvement, innovation in medical education, and the provision of high-quality clinical and educational resources. It also sought to strengthen and diversify the healthcare workforce to better meet community healthcare needs.

Over the past 15 years, the program has become an important component of family medicine training and workforce development in the northern emirates. However, it has also encountered challenges related to resource allocation, faculty retention, resident workload, and alignment with broader healthcare reforms. As such, a comprehensive review of this 15-year experience is warranted to identify what has worked, what needs improvement, and what lessons can be shared with other health systems facing similar circumstances.

This study aimed to evaluate the evolution of the EHS FM Residency Program over a 15-year period and to examine participants’ perceived educational outcomes, including satisfaction with the program, perceived clinical competency, and preparedness for independent practice. In addition, the study sought to identify strengths, challenges, and opportunities for program and curriculum improvement.

## Materials and methods

Study design

This study employed a sequential explanatory mixed-methods design to evaluate the evolution and perceived educational outcomes of the EHS Family Medicine Residency Program over a 15-year period. Quantitative data were initially collected through a structured survey administered to program alumni and current fourth-year residents (R4). Subsequently, qualitative data were obtained through focus group discussions involving purposively selected residents, alumni, and faculty members. Quantitative and qualitative data were analyzed separately and integrated during interpretation to provide a comprehensive understanding of program outcomes and developmental changes.

Study setting

The study was conducted within the EHS Family Medicine Residency Program in the United Arab Emirates. The program is a four-year postgraduate training program delivered across 10 primary healthcare centers and seven secondary and tertiary healthcare institutions within the northern emirates.

Study participants and sampling

Quantitative Component

The target population included all program alumni and current R4 residents eligible at the time of data collection. The eligible population consisted of 71 individuals, including 63 alumni and eight current R4 residents. A total of 64 participants completed the survey and were included in the final quantitative analysis, yielding a response rate of 90.1%.

The survey collected demographic and professional characteristics, including age, gender, nationality, program status, current clinical practice type, and postgraduate qualifications.

Qualitative Component

Participants were purposively selected to ensure representation of key stakeholders, including faculty members, current R4 residents, alumni, and alumni who subsequently assumed faculty positions. This approach ensured diversity in roles and years of post-graduation experience, enabling the inclusion of multiple perspectives on program development and outcomes. A semi-structured interview guide was independently developed by two research members, and consensus among researchers was achieved regarding the final guide. Two focus group discussions were conducted, involving a total of 11 participants. The first group included five participants, while the second included six participants.

Survey instrument and data collection

The survey instrument was developed by the research team based on a review of relevant literature on residency program evaluation and the program’s educational objectives. Survey items were designed to reflect core domains relevant to family medicine residency training, including the educational environment, supervision, perceived competency, preparedness for practice, and professional development. Content validity was assessed through review by experienced faculty members involved in postgraduate medical education and family medicine training. Prior to implementation, the instrument was pilot tested among five family physicians actively involved in postgraduate medical education and family medicine training to assess the clarity, wording, and ease of survey completion. Based on their feedback, minor modifications were made to wording and question formatting. Because the instrument was developed primarily for exploratory program evaluation rather than psychometric scale development, formal validation and reliability testing were not performed. The survey consisted of five sections covering participants’ demographic and professional characteristics, satisfaction with residency program components, perceived clinical competency, preparedness for independent clinical practice, and open-text responses exploring recommendations, strengths, and areas for program improvement. The complete survey instrument is provided in Appendix 1.

Responses were recorded using five-point Likert scales evaluating satisfaction, confidence, and preparedness across multiple educational and clinical domains, including clinical training, academic activities, faculty supervision, communication skills, and practice management.

An electronic informed consent form and survey link were distributed to all eligible participants by email and WhatsApp. Participants who consented electronically were automatically directed to the survey.

Focus group data collection

Qualitative data were collected through two in-person focus group discussions, each lasting approximately one hour. Discussions were conducted in English and moderated by a member of the research team familiar with the residency program context using a semi-structured interview guide.

Discussion topics explored curriculum evolution, educational and clinical training experiences, assessment methods, supervision, program strengths, ongoing challenges, and recommendations for future program improvement. The semi-structured focus group discussion guide is provided in Appendix 2.

All discussions were audio-recorded with participant consent and transcribed verbatim for analysis. After the second focus group discussion, no substantially new themes emerged, and the research team considered the collected data sufficient to address the study objectives.

Quantitative data analysis

Quantitative data were analyzed using the Statistical Analysis System (SAS) Viya 3.5 software (SAS Inc., Cary, NC, USA). Composite domain scores for satisfaction, perceived competency, and preparedness were calculated by averaging responses to related Likert-scale items within each domain. Because the resulting distributions demonstrated clustering toward higher values and limited variability, analyses focused primarily on median composite scores, ranges, item-level distributions, and non-parametric comparisons.

Categorical variables were summarized using frequencies and percentages. Composite domain scores were summarized using medians and ranges.

Given the ordinal nature of the data and evidence of ceiling effects, non-parametric statistical tests were used for subgroup comparisons. Wilcoxon rank-sum tests were applied for two-group comparisons, whereas Kruskal-Wallis tests were used for comparisons involving more than two groups. Post-hoc pairwise comparisons were conducted where applicable because subgroup analyses were exploratory and several subgroup categories were relatively small; p-values were interpreted cautiously. Spearman's rank correlation analysis was performed to examine associations between satisfaction, perceived competency, and preparedness scores. Because of subgroup imbalance and limited sample size in certain categories, particularly gender comparisons, subgroup findings were interpreted cautiously and considered exploratory.

Qualitative data analysis

Qualitative data were analyzed using inductive thematic analysis. Two research team members independently reviewed the transcripts and conducted initial coding. Codes were iteratively refined, organized into categories, and subsequently grouped into broader themes representing participants’ perceptions of program evolution, educational strengths, and areas for improvement. Consensus regarding the final thematic framework was achieved through iterative discussion among the research team.

To enhance analytical rigor and trustworthiness, investigator triangulation and reflexive analytical discussions were used to improve credibility and minimize interpretive bias. Two members of the research team held leadership roles within the residency program; therefore, reflexive discussions were incorporated throughout the analytical process to ensure consistency between emerging themes and participant narratives and to reduce potential institutional bias. Representative quotations were selected to enhance transparency and illustrate key themes. Coding and thematic organization were conducted manually. Integration of quantitative and qualitative findings occurred at the interpretation stage.

Ethical considerations

Ethical approval was obtained from the UAE Ministry of Health and Prevention Research Ethics Committee (Approval Reference No.: MOHAP/DXB-REC/J.J.J /No. 135/ 2025). Participation in both the survey and focus group discussions was voluntary. Electronic informed consent was obtained from all participants prior to participation. All data were anonymized before analysis to ensure confidentiality.

## Results

Participant characteristics

Of 71 eligible individuals, 64 completed the survey and were included in the quantitative analysis, yielding a response rate of 90.1%. Respondents included 56 alumni and eight current fourth-year residents. Two focus group discussions were also conducted with 11 purposively selected participants.

Most survey respondents were female (59/64, 92.2%) and UAE nationals (50/64, 78.1%). The largest age group was ≥40 years (24/64, 37.5%), followed by ≤34 years (23/64, 35.9%) and 35-39 years (17/64, 26.6%). Among alumni, the most frequently reported time since residency completion was 1-3 years (26.8%), followed by 7-10 years (23.2%), more than 10 years (21.4%), 4-6 years (19.6%), and less than 1 year (8.9%). Primary healthcare was the most frequently reported practice setting (45/64, 70.3%); as multiple practice roles could be selected, percentages exceeded 100%. Participant characteristics are summarized in Table 1.

**Table 1:** Demographic and professional characteristics of participants (N = 64). * Applicable only to alumni respondents (n=56). ** Multiple selections were allowed; therefore, percentages may exceed 100.0%. *** Fellowship or other academic qualifications are reported as a separate descriptive category based on free-text responses. UAE, United Arab Emirates; R4, fourth-year resident; MRCGP, Member of the Royal College of General Practitioners.

Characteristic	Category	n (%)
Age group	≤34 years	23 (35.9)
	35–39 years	17 (26.6)
	≥40 years	24 (37.5)
Gender	Female	59 (92.2)
	Male	5 (7.8)
Nationality	UAE	50 (78.1)
	Other	14 (21.9)
Program status	Alumni	56 (87.5)
	R4 Resident	8 (12.5)
Time since residency completion*	<1 year	5 (8.9)
	1–3 years	15 (26.8)
	4–6 years	11 (19.6)
	7–10 years	13 (23.2)
	>10 years	12 (21.4)
Board certifications*	Arab Board only	34 (60.7)
	Arab Board and Emirati Board	9 (16.1)
	Arab Board and MRCGP	7 (12.5)
	Emirati Board only	4 (7.1)
	In process	2 (3.6)
Current practice setting**	Primary healthcare	45 (70.3)
	Leadership/administrative role	19 (29.7)
	Academic/teaching role	17 (26.6)
	Hospital-based clinical care	14 (21.9)
	Currently not practicing	7 (10.9)
	Community/public health care	7 (10.9)
Fellowship or other academic qualifications***	Yes	8 (12.5)

Satisfaction with the residency program

The median satisfaction score was 4.55 out of 5.00 (range, 3.10-5.00). Overall, 38 respondents (59.4%) were very satisfied, 23 (35.9%) were satisfied, two (3.1%) were neutral, and one (1.6%) was very dissatisfied. All respondents indicated that they would recommend the program.

Faculty feedback and supervision received the highest ratings, with 43 respondents (67.2%) rating this component as excellent and 21 (32.8%) as good. Faculty teaching was rated excellent by 41 respondents (64.1%) and good by 23 (35.9%). Academic day activities were rated excellent by 42 respondents (65.6%), good by 21 (32.8%), and fair by one (1.6%). Hospital-based clinical training showed comparatively greater variability, with 21 respondents (32.8%) rating it excellent, 34 (53.1%) good, and nine (14.1%) fair. Work-life balance was rated excellent by 23 respondents (35.9%), good by 33 (51.6%), and fair by eight (12.5%). Similar concerns regarding workload and work-life balance also emerged in optional open-text comments.

Perceived clinical competency

The median perceived competency score was 4.36 (range, 3.53-5.00). Composite perceived competency scores were generally high across respondents, although variability was observed at the item level. Confidence was highest for communication and interpersonal skills, with 47 respondents (73.4%) reporting being extremely confident and 17 (26.6%) confident. Outpatient care was also highly rated, with 38 respondents (59.4%) extremely confident and 26 (40.6%) confident. Greater variability was observed for inpatient adult care, where 12 respondents (18.8%) were extremely confident, 36 (56.3%) confident, 13 (20.3%) somewhat confident, and three (4.7%) minimally confident. Behavioral and mental health also showed variability, with 17 respondents (26.6%) extremely confident, 32 (50.0%) confident, and 15 (23.4%) somewhat confident.

Preparedness for independent practice

The median preparedness score was 4.37 (range, 3.12-5.00). Overall, 33 respondents (51.6%) reported being extremely well prepared, 29 (45.3%) very well prepared, and two (3.1%) somewhat prepared for independent family medicine practice. Preparedness was strongest for patient-centered communication and cultural competence, where 37 respondents (57.8%) reported being extremely well prepared and 26 (40.6%) very well prepared. Time management and patient flow showed comparatively greater variability, with 28 respondents (43.8%) extremely well prepared, 32 (50.0%) very well prepared, and four (6.3%) somewhat prepared. Selected item-level outcomes and composite domain scores for satisfaction, perceived competency, and preparedness for practice are summarized in Table 2.

**Table 2:** Selected item-level outcomes and domain scores for satisfaction, perceived competency, and preparedness for practice (N = 64). * Work-life balance refers to the program’s effectiveness in promoting wellness, preventing burnout, managing work hours, and allowing time for personal and family life. Domain scores were calculated by averaging Likert-scale responses within each domain.

Variable	Highest-scale response n (%)	Second highest response category n (%)	Other responses n (%)
Satisfaction Outcomes			
Overall satisfaction	Very satisfied: 38 (59.4)	Satisfied: 23 (35.9)	Neutral/very dissatisfied: 3 (4.7)
Academic day activities	Excellent: 42 (65.6)	Good: 21 (32.8)	Fair: 1 (1.6)
Faculty teaching	Excellent: 41 (64.1)	Good: 23 (35.9)	Fair/Poor/Very poor: 0 (0)
Faculty supervision	Excellent: 43 (67.2)	Good: 21 (32.8)	Fair/Poor/Very poor: 0 (0)
Work-life balance*	Excellent: 23 (35.9)	Good: 33 (51.6)	Fair: 8 (12.5)
Median satisfaction score: 4.55 (Range: 3.10-5.00)			
Perceived Competency Outcomes			
Outpatient care	Extremely confident: 38 (59.4)	Confident: 26 (40.6)	Somewhat/minimally confident: 0 (0)
Chronic disease management	Extremely confident: 30 (46.9)	Confident: 32 (50.0)	Somewhat confident: 2 (3.1)
Communication skills	Extremely confident: 47 (73.4)	Confident: 17 (26.6)	Somewhat/minimally confident: 0 (0)
Median competency score: 4.36 (Range: 3.53-5.00)			
Preparedness for Independent Practice			
Overall preparedness	Extremely well prepared: 33 (51.6)	Very well prepared: 29 (45.3)	Somewhat prepared: 2 (3.1)
Clinical decision-making	Extremely well prepared: 30 (46.9)	Very well prepared: 33 (51.6)	Somewhat prepared: 1 (1.6)
Time management and patient flow	Extremely well prepared: 28 (43.8)	Very well prepared: 32 (50.0)	Somewhat prepared: 4 (6.3)
Median preparedness score: 4.37 (Range: 3.12-5.00)			

Subgroup comparisons

Because domain scores were ordinal-derived and showed ceiling effects, subgroup comparisons were conducted using non-parametric tests and interpreted as exploratory. Female respondents had higher satisfaction scores than male respondents (median 4.60 vs. 4.00; p<0.01), although this comparison is limited by the very small number of male respondents. Satisfaction did not differ significantly by age group, nationality, or program status.

Among alumni, satisfaction, perceived competency, and preparedness differed by time since residency completion. Alumni who had completed training more than 10 years earlier reported the highest satisfaction score (median 4.90), whereas those 1-3 years post-residency reported the lowest score (median 4.20; p = 0.001). Perceived competency also differed by post-residency duration (p = 0.01), with higher median scores among alumni >10 years post-residency than those 1-3 years post-residency. Preparedness differed by post-residency duration (p = 0.0037), with higher scores among alumni >10 years post-residency compared with those 1-3 years and 7-10 years post-residency.

Comparisons of satisfaction, perceived competency, and preparedness scores across demographic and training-related subgroups are presented in Table 3.

**Table 3:** Comparison of satisfaction, perceived competency, and preparedness scores across demographic groups (N = 64). p-values were calculated using Wilcoxon rank-sum or Kruskal-Wallis tests, as appropriate. * Applicable only to alumni respondents (n = 56). R4, fourth-year resident.

Variable	Group	Satisfaction median	p-value	Competency median	p-value	Preparedness median
Gender	Female (n = 59)	4.60	<0.01	4.40	0.57	4.38
	Male (n = 5)	4.00		4.20		4.38
Age group	≤34 years (n = 23)	4.50	0.57	4.27	0.25	4.13
	35–39 years (n = 17)	4.60		4.47		4.63
	≥40 years (n = 24)	4.65		4.47		4.63
Program status	Program alumni (n = 56)	4.60	0.24	4.40	0.26	4.44
	R4 resident (n = 8)	4.40		4.30		4.06
Post-residency duration*	<1 year (n = 5)	4.60	0.001	4.47	0.01	4.63
	1–3 years (n = 15)	4.20		4.20		4.13
	4–6 years (n = 11)	4.70		4.40		4.75
	7–10 years (n = 13)	4.10		4.07		4.13
	>10 years (n = 12)	4.90		4.80		4.94

Correlations between outcome domains

Satisfaction was positively correlated with perceived competency (Spearman ρ=0.60, p<0.0001) and preparedness for independent practice (ρ=0.64, p<0.0001). These correlations indicate moderate positive associations and should not be interpreted causally. Spearman correlation analyses between satisfaction and the outcome domains are presented in Table 4.

**Table 4:** Correlations between satisfaction, perceived competency, and preparedness scores (N = 64).

Outcome pair	Spearman ρ	p-value
Satisfaction vs. perceived competency	0.60	<0.0001
Satisfaction vs. preparedness for practice	0.64	<0.0001

Qualitative results

Optional open-text survey responses were reviewed descriptively and considered alongside focus group findings to contextualize participants’ perceptions of program strengths and areas for improvement.

Qualitative findings suggested a clear trajectory of perceived program evolution toward a more structured, learner-centered training model incorporating competency-based educational principles.

Participants consistently described progressive curriculum transformation from a less defined structure to more focused learning objectives. This shift was further reinforced by the alignment of curriculum content with the scope of family medicine practice, particularly through the inclusion of relevant subspecialties, including geriatrics and practice management, and clearer rotation objectives.

The integration of academic and clinical learning emerged as a central feature of the program. Participants highlighted the role of the academic day as a cornerstone of learning. The alignment of academic sessions with clinical exposure was perceived to enhance understanding and retention.

Teaching approaches have also evolved toward more interactive and self-directed methods. Residents described increased opportunities for peer teaching. Similarly, residents frequently described outpatient settings as more educationally valuable than inpatient settings. Participants also described the use of institutional digital learning platforms that enabled access to educational resources and supported self-directed learning.

Assessment practices were described as having undergone substantial transformation, shifting from predominantly summative approaches to continuous, competency-based, and multimodal evaluation methods. Residents described these assessment approaches as useful for monitoring progress, identifying learning needs, and the preparedness for independent practice. The introduction of digital platforms further supported this shift, enabling more efficient tracking of progress and documentation.

Supervision was generally perceived as supportive, particularly within primary health care settings, where participants highlighted the accessibility and approachability of supervisors. This supportive environment fostered progressive resident autonomy, reflected in the experience of managing cases independently before discussing them subsequently with supervisors. However, supervision experiences were not uniform, with participants reporting notable variability across hospital rotations. Participants noted that supervision often depended on the specific hospital rotation, with some settings providing more consistent support than others, highlighting inconsistencies that may influence the quality of learning experiences and educational support.

Participants also described substantial improvements in resident competencies and professional development over time. Participants perceived residents as increasingly knowledgeable, confident, and well prepared for independent practice. Beyond clinical competency, the program was viewed as promoting research engagement, leadership development, and broader professional growth, all of which contributed to strengthening professional identity and self-confidence.

The program’s strengths were further reflected in its growing reputation and achievements. Participants highlighted accreditation by the Emirati Board as a major milestone, alongside the supportive learning environment and the opportunities provided for professional and career development. The introduction of a digital residency management system was also perceived as a significant advancement in program organization and training processes.

Despite these positive developments, participants identified ongoing challenges. A recurrent issue was the tension between clinical workload and educational activities. Additionally, administrative demands and documentation requirements were also described as contributing to reduced teaching time and increased workload.

Overall, participants described the program as having undergone substantial and continuous development, with notable strengths in curriculum structure, educational innovation, assessment systems, supervision, and resident development, while also identifying persistent operational and workload-related challenges requiring further improvement.

The major qualitative themes, subthemes, and representative participant quotations are summarized in Table 5.

**Table 5:** Key qualitative themes, subthemes, and representative participant quotations.

Theme	Sub-theme	Example quotations
Curriculum evolution	Improved alignment with family medicine practice	“The curriculum is now much more focused and clearer… residents know exactly what they need to cover...” (Alumni/faculty participant) “More subspecialty exposure that is relevant to family medicine, such as lactation clinics and geriatric care...” (Alumni/faculty participant)
	Curriculum expansion	“Research was not included at the beginning, but later research and audits were added.” (Alumni participant) “It is very detailed and well structured, guiding us on what to follow and what to complete.” (R4 resident)
	Academic-clinical integration	“The academic day agenda is now aligned with what residents are seeing clinically...” (Alumni/faculty participant)
Learner-centered teaching and educational innovation	Shift to active, learner-centered learning	“Residents started presenting to each other and leading discussions… increased confidence.” (Alumni participant)
	Academic day as a core learning platform	“I think the academic day is the most effective teaching method.” (R4 resident)
	Outpatient-focused learning	“I learned much more from outpatient settings than inpatient settings...” (R4 resident)
	Case-based and interactive learning	“I learned more from role play, especially during the first two years.” (R4 resident)
	Peer learning and collaborative culture	“Yes…, and another important aspect is the culture of peer learning..” (Alumni participant)
	Self-directed and digital learning	“Everything is available to them [through online platforms] … access to updated knowledge and resources.” (Faculty participant)
Evolving assessment & evaluation	Continuous assessment	“Previously assessment was mainly at the end… now it is continuous.” (Alumni participant)
	Assessment for learning and improvement	“Assessment now helps residents to identify their weaknesses.” (Faculty participant) “At the beginning it felt overwhelming… but later we realized it was helpful.” (R4 resident)
	Tracking progress and performance	“We know how much done and how much is remaining… we can focus on other procedures.” (R4 resident) “Now it is easy to send a request through the system electronically and monitor the record.” (Alumni/faculty participant)
	Multimodal assessments	“…also, the assessment from different faculty members was useful and added value” (R4 resident) “…now we have many different assessment methods” (Faculty participant)
Supervision and progressive resident autonomy	Supportive supervision in the primary care setting	“If you have questions, you can easily reach your supervisor.” (Alumni participant)
		“In PHC settings supervision was excellent.” (Alumni participant)
	Variability across hospital rotations	“Supervision depends on the rotation… hospital supervision varies more.” (R4 resident)
	Structured supervision and monitoring	“We are monitoring … each resident’s patient load through the HIM report to ensure good exposure to all services as per curriculum” (Faculty participant)
	Increased resident’s independence	“I am able to sit alone, manage cases independently, and then discuss them later.” (R4 resident)
Resident development	Improved clinical competency	“Residents today are more knowledgeable and more up to date.” (Faculty participant) “…by year three they can see patients independently.” (Faculty participant) “I am able to sit alone and manage cases independently; then I will discuss cases later...this builds my confidence.” (R4 resident)
	Scholarly and community engagement	“Residents now present audits and research projects at conferences.” (Alumni-/faculty participant) “..we have been involved in giving presentations to GP physicians...more confident...inspiring educational experience.” (R4 resident) “…also participate in community engagement activities” (Alumni/faculty participant)
	Leadership and career progression	“Many graduates… have progressed into leadership positions.” (Alumni-faculty participant)
Program outcomes and impact	Perceived graduate development and career progression	“Very big difference… doctors are more knowledgeable and up to date.” (Faculty participant) “Many graduates from the program are now in leadership roles…. others continue postgraduate education or subspecialty training.” (Alumni/faculty participant)
	Accreditation and recognition	“Being Emirati Board accredited is also another big achievement.” (Alumni/faculty participant) “Many doctors… ask about how to apply… growing recognition.” (Faculty participant)
	Supportive learning environment	“There is a supportive environment… a strong professional community.” (Alumni participant) “…also, residents have many opportunities to attend workshops and conferences, … helps their professional development.” (Faculty participant)
	Digitization of residency management	“New Innovations electronic systems… very useful and efficient.” (R4 resident) “….it is very useful and efficient compared to manual records.” (R4 resident)
	Active community engagement	“.. residents are now involved in non-academic professional activities...and community participation.” (Alumni/faculty participant)
Operational and educational challenges	Clinical and educational work balance	“Busy clinics… difficult to balance service, teaching, and assessment.” (Faculty participant) “Discussions with supervisors sometimes become brief due to time constraints.” (Alumni/faculty participant)
	Limited educational engagement	“Some departments focus more on completing notes rather than teaching.” (R4 resident) “Sometimes there are not enough faculty members.” (Faculty participant)
	Administrative burden	Yes, documentation requirements and workload have increased…reduces the time available for teaching.” (Faculty participant)

## Discussion

This mixed-methods evaluation identified substantial participant-perceived evolution of the EHS Family Medicine Residency Program over the past 15 years toward a more structured, learner-centered, and competency-oriented training model, with participants reporting high levels of satisfaction, perceived competency, and preparedness for independent practice. The integration of quantitative and qualitative findings suggests progressive alignment of the program’s educational structure with principles of competency-based medical education and the evolving needs of family medicine practice.

Overall satisfaction with the program was high, particularly regarding faculty teaching, supervision, and academic day activities. These findings were reinforced qualitatively, where participants described the academic day as a central and protected learning platform that effectively integrated curriculum content with clinical exposure. The strong ratings for faculty supervision and teaching likely reflect the importance of structured educational support and positive learner-supervisor relationships within residency training. These findings are consistent with literature demonstrating that supportive learning environments, accessible supervision, and constructive feedback are important components of postgraduate workplace learning and may contribute to reflective learning and perceived competency development in residency training [9].

Participants reported high levels of perceived competency and preparedness for independent practice, particularly in communication skills, outpatient care, chronic disease management, and patient-centered practice. These findings may reflect the program’s strong emphasis on primary healthcare-based training, continuity of care, and longitudinal patient relationships within ambulatory settings. Qualitative findings further demonstrated that residents highly valued outpatient experiential learning and perceived it as more educationally relevant than inpatient exposure for future family medicine practice. This observation is consistent with the core orientation of family medicine training toward community-based, continuity-focused, and patient-centered care, in which ambulatory clinical exposure plays a central role in the development of comprehensive primary care competencies [2, 10]. The perceived educational value of outpatient training may also reflect the closer alignment between ambulatory continuity experiences and the real-world scope of family medicine practice compared with fragmented subspecialty inpatient exposure.

The qualitative findings demonstrated substantial curriculum transformation over time, including clearer learning objectives, improved alignment with family medicine practice, incorporation of relevant subspecialties, and expansion of scholarly activities such as research and audit participation. Participants also described a progressive shift toward learner-centered and interactive teaching approaches, including peer teaching, role play, case-based learning, and self-directed digital learning. Together, these findings suggest progressive alignment of the program with contemporary residency education frameworks that emphasize active learning, reflective practice, self-directed learning, and competency development [11, 12].

Assessment systems were also perceived to have evolved substantially from predominantly summative evaluation toward continuous, multimodal, and competency-based assessment approaches. Contemporary competency-based medical education (CBME) frameworks emphasize progressive competency acquisition, workplace-based assessment, feedback-informed learning, and learner development within authentic clinical environments [13, 14]. This evolution reflects a broader international movement toward programmatic assessment models, which emphasize longitudinal evidence gathering, workplace-based assessment, feedback integration, and progressive entrustment rather than reliance on isolated summative examinations [15, 16]. Within the program, participants described the use of multiple complementary assessment approaches, including workplace-based assessments such as case-based discussions (CBD), Direct Observation of Procedural Skills (DOPS), and Mini-Clinical Evaluation Exercises (Mini-CEX), which have increasingly been recognized as important tools for formative assessment and competency development in postgraduate medical education [17]. In addition to structured internal Objective Structured Clinical Examination (OSCE) assessments, annual in-training written examinations are conducted in collaboration with the American Board of Family Medicine, midpoint and end-of-rotation evaluations, academic day assessments, and audit or scholarly activity evaluations. Although residents initially perceived some assessment requirements as burdensome, they later acknowledged their value in identifying learning gaps, monitoring progress, and supporting preparedness for independent practice. The introduction of digital educational and residency management systems appeared to further facilitate this transition by improving documentation, feedback processes, and longitudinal performance tracking. Similar transitions toward competency-oriented and workplace-based assessment systems have increasingly been described across postgraduate medical education internationally [15, 16].

Supervision emerged as an important contributor to resident development, particularly within primary healthcare settings where participants described supervisors as highly accessible and supportive. This educational environment appeared to facilitate progressive resident autonomy, reflective learning, and confidence in independent case management. However, participants also identified variability in supervision quality across hospital rotations, suggesting inconsistency in educational experiences between training sites. Similar variability in supervision and workplace learning experiences has been described across postgraduate medical education and may relate to differences in faculty engagement, competing service demands, institutional culture, and the extent to which educational responsibilities are protected within clinical environments [9, 18]. In addition, the stronger perceived educational value of ambulatory supervision may reflect the closer alignment between continuity-based primary care training and the real-world scope of family medicine practice compared with subspecialty-focused inpatient rotations.

Beyond clinical competency, participants highlighted broader professional development outcomes, including research engagement, leadership development, teaching participation, and community involvement. Participants described several graduates as progressing into leadership positions, academic roles, and further postgraduate training. These findings suggest that the program may contribute not only to the development of the family medicine workforce but also to broader academic and professional capacity building within the UAE primary healthcare system. Accreditation by the Emirati Board was also perceived by participants as an important milestone that enhanced program recognition, educational standardization, and professional identity.

Despite these positive findings, participants identified persistent operational challenges, particularly balancing clinical service demands with educational activities. Workload pressures, administrative documentation requirements, and limited protected teaching time were perceived as barriers to optimal educational engagement. Similar challenges have been described internationally across residency training programs, where high workload, administrative burden, and insufficient protected educational time have been associated with resident burnout, reduced well-being, and lower educational satisfaction [19, 20]. Addressing these operational challenges may therefore be necessary to sustain educational quality, resident well-being, and long-term program sustainability. These findings may be particularly relevant within the UAE healthcare context, where strengthening the national primary healthcare workforce and expanding family medicine training capacity remain strategic healthcare priorities.

Interestingly, alumni who completed training more than 10 years earlier reported higher satisfaction, perceived competency, and preparedness scores than more recent graduates. Several explanations may account for this finding, including increased professional confidence over time, career progression, retrospective appraisal of training experiences, recall effects, or cohort-related differences in training structure, assessment requirements, and expectations across different stages of program development. However, these subgroup findings should be interpreted cautiously, as several comparisons involved relatively small sample sizes and exploratory analyses that were not designed to establish causal or longitudinal relationships.

Overall, the findings suggest that the EHS Family Medicine Residency Program has undergone substantial and sustained development over the past 15 years and demonstrates several participant-perceived characteristics aligned with contemporary competency-based residency education models. Participants generally perceived the program as contributing positively to preparedness for independent practice, professional development, and engagement in academic and leadership activities. Collectively, these findings suggest that the program may contribute to strengthening the family medicine workforce and supporting the evolving primary healthcare needs of the UAE. Although the findings reflect the experience of a single residency program, several educational features identified in this study may offer insights for residency programs within the UAE, the Gulf region, and other settings undergoing similar healthcare and educational transformation. Differences in healthcare systems and training structures, however, should be considered when interpreting the transferability of these findings.

Recommendations

Future program development should focus on strengthening the consistency and quality of supervision across training sites through structured faculty development initiatives, clearer educational expectations, and protection of dedicated teaching responsibilities within both primary healthcare and hospital settings. Additional efforts to optimize workload distribution, reduce unnecessary administrative burden, and preserve protected educational time may further enhance resident well-being, educational engagement, and learning quality.

Continued investment in learner-centered educational strategies, ambulatory continuity-based training, academic-clinical integration, digital educational systems, and competency-based assessment approaches is recommended to support continued educational quality improvement and competency-based training development. Further refinement of workplace-based assessment processes, longitudinal feedback systems, and programmatic assessment frameworks may also strengthen competency development and reflective learning within the residency program.

Future multicenter and longitudinal research incorporating objective measures of clinical performance, board examination performance, workforce retention, career progression, academic productivity, patient care outcomes, and external competency assessments is recommended to further evaluate the long-term impact of family medicine residency training within the UAE and the broader Gulf-region healthcare context.

Limitations

This study has several limitations. First, it was conducted within a single-family medicine residency program in the UAE, which may limit the generalizability and transferability of the findings to other residency programs and healthcare settings. Second, the study relied primarily on self-reported measures of satisfaction, perceived competency, and preparedness for independent practice, which may be influenced by recall bias, social desirability bias, and variability in self-assessment. Given the program-based nature of the study, these factors may have contributed to more favorable evaluations of educational experiences and outcomes. In addition, the clustering of responses toward higher ratings suggests possible ceiling effects that may have reduced variability and limited discrimination across some outcome domains. Although the mixed-methods design enhanced contextual understanding and methodological triangulation, the qualitative findings reflected the perspectives of purposively selected participants and therefore may not fully represent the experiences of all residents, alumni, or faculty members within the program. Furthermore, the study did not include objective measures of clinical performance, patient outcomes, external competency assessments, or workforce outcomes such as board examination performance, workforce retention, and verified leadership trajectories. Consequently, the findings primarily reflect participant perceptions rather than independently verified educational or workforce outcomes.

## Conclusions

The EHS Family Medicine Residency Program has undergone substantial development over the past 15 years toward a more structured and competency-oriented training model. Participants generally perceived the program positively with respect to supervision, educational experiences, preparedness for independent practice, and professional development. Despite ongoing operational and workload-related challenges, the findings suggest that the program may contribute to strengthening the family medicine workforce and supporting evolving primary healthcare needs within the UAE.

## Appendices

Appendix 1 

**Table 6:** Participant survey instrument. The questionnaire was developed by the study investigators based on a literature review and program evaluation objectives, followed by expert review for content validity and pilot testing for clarity and relevance. EHS, Emirates Health Services; MRCGP, Member of the Royal College of General Practitioners.

Section	Item no.	Question/variable	Response format	Notes
Section 1: Demographic and Background Information	1	Age group	≤30; 30-34; 35-39; 40-44; 45-49; ≥50	Single response
	2	Gender	Male; female	
	3	Nationality	UAE; GCC; Others	
	4	What is your role in the program?	R4 Resident; Program alumni	
	5.1	How long has it been since you completed your residency training?	<1 year; 1–3; 4–6; 7–10; >10 years	Alumni only; Single response
	5.2	What type(s) of clinical practice are you engaged in? (Select all that apply)	Primary healthcare; Hospital-based clinical care; Community/public health care; Academic/teaching role; Administrative/leadership role; Currently not practicing	Alumni only; multiple response
	5.3	Board / Fellowship Certifications	Arab Board; Emirati Board; MRCGP; Fellowship / other academic qualifications (specify); In process (specify); None	
Section 2: Satisfaction with the Residency Program Components	2.1	Overall, how satisfied are you with the EHS Family Medicine Residency Program?	Very satisfied; Satisfied; Neutral; Dissatisfied; Very dissatisfied	Single response
	2.2	How well does the program foster a supportive learning environment?	Excellent; Good; Fair; Poor; Very Poor	
	2.3	Please rate the quality of the following components:	1=Very Poor (ineffective, poorly organized); 2=Poor (major shortcomings); 3=Fair (basic, inconsistent); 4=Good (meets expectations); 5=Excellent (highly effective, well-structured)	If satisfaction is rated ≤ 3, explain how this component affected your preparedness for practice [Open text box]
	2.3.1	Clinical training in the primary healthcare setting		
	2.3.2	Clinical training in the secondary (hospital-based) healthcare setting		
	2.3.3	Academic day activities		
	2.3.4	Scholarly activities and training (include research, clinical audits and journal club)		
	2.3.5	Faculty teaching		
	2.3.6	Faculty feedback and supervision		
	2.3.7	Availability of resources: the accessibility and quality of educational tools (e.g., library, electronic resources, simulation labs)		
	2.3.8	Work-life balance: The program’s effectiveness in promoting wellness, preventing burnout, managing work hours, and allowing time for personal and family life		
	2.4	Would you recommend this program to others?	Yes; No; Maybe	Single response
Section 3: Perceived Competency (Self-Assessment)	3	Please rate your level of confidence in performing the following competencies based on your training:	1=Not confident; 2=Minimally confident; 3=Somewhat confident; 4=Confident; 5=Extremely confident	Single response
	3A	Core Clinical Competence		
	3A.1	Outpatient care		
	3A.2	Inpatient adult care		
	3A.3	Pediatric care		
	3A.4	Women’s health		
	3A.5	Geriatric care		
	3A.6	Emergency/urgent care		
	3A.7	Chronic disease management (e.g., diabetes, hypertension, asthma)		
	3A.8	Behavioural and mental health		
	3A.9	Common primary healthcare procedures		
	3B	Preventive Health		
	3B.1	Preventive care and screening		
	3B.2	Health promotion and patient education		
	3C	Professional and Communication Skills		
	3C.1	Communication and interpersonal skills		
	3C.2	Teamwork and interprofessional collaboration		
	3C.3	Systems-based practice and use of resources		
	3C.4	Evidence-based medicine and clinical decision-making		
Section 4: Preparedness for Practice	4.1	Overall, how well did the EHS residency program prepare you for independent family medicine practice?	Extremely well; Very well; Somewhat well; Not very well; Not at all	Single response
	4.2	How prepared do you feel in the following areas of real-world practice:	1=Not at all prepared; 2=Minimally prepared; 3=Somewhat prepared; 4=Well prepared; 5=Very well prepared	If preparedness is rated ≤ 3, explain how this area affected your preparedness for practice [Open text]
	4.2.1	Clinical decision-making		
	4.2.2	Managing multiple comorbidities		
	4.2.3	Time management and patient flow		
	4.2.4	Practice management		
	4.2.5	Work-life balance and resilience		
	4.2.6	Patient-centered communication and cultural competence		
	4.2.7	Supervising junior colleagues		
Section 5: Overall Recommendations	5.1	What are the program's greatest strengths?	Open text	Optional
	5.2	What areas most need improvement?	Open text	Optional

Appendix 2 

**Table 7:** Focus group discussion guide.

No.	Topics	Guiding questions
1	Curriculum evolution and training methods	How has the curriculum changed over the past 15 years?
		Which teaching or training methods have been most effective or least effective?
		How have residents' performance assessment, clinical teaching, and supervision evolved?
2	Program successes and challenges	What have been the most significant achievements of the program?
		What challenges has the program encountered, and how were they addressed?
3	Recommendations for improvement	What improvements would you recommend for the curriculum or program structure?
		How can the training experience be strengthened for future residents?
